# Supplementary material for: Optoelectronic properties and ultrafast carrier dynamics of copper iodide thin films
Source: Nat Commun. 2022 Oct 26;13:6346. doi: 10.1038/s41467-022-34117-8 (PMC9606309; doi:10.1038/s41467-022-34117-8)
Supplement: Supplementary file 1 — Supplementary Information [file 41467_2022_34117_MOESM1_ESM.pdf]

Supplementary Information

Optoelectronic properties and ultrafast carrier dynamics of copper iodide thin films

Zhan Hua Li,[1,2] Jia Xing He,[3] Xiao Hu Lv,[1,2] Ling Fei Chi,[1] Kingsley O. Egbo,[4,5] Ming-De Li,[3,*] Tooru Tanaka,[6] Qi Xin Guo,[6] Kin Man Yu,[4] and Chao Ping Liu[1,2*]

[1] Research Center for Advanced Optics and Photoelectronics, Department of Physics, College of Science, Shantou University, Shantou, Guangdong 515063, China

[2] Center of Semiconductor Materials and Devices, Shantou University, Shantou, Guangdong 515063, China

[3] Department of Chemistry and Key Laboratory for Preparation and Application of Ordered Structural Materials of Guangdong Province, Shantou University, Shantou, Guangdong 515063, China

[4] Department of Physics, City University of Hong Kong, 83 Tat Chee Ave., Kowloon, Hong Kong

[5] Paul-Drude-Institut fur Festkorperelektronik, Liebniz-Institut im Forschungsverbund Berlin e. V., Hausvogteiplatz 5-7, 10117 Berlin, Germany

[6] Department of Electrical and Electronic Engineering, Synchrotron Light Application Center, Saga University, Saga 840-8502, Japan

* Corresponding: mdli@stu.edu.cn; cpliu@stu.edu.cn

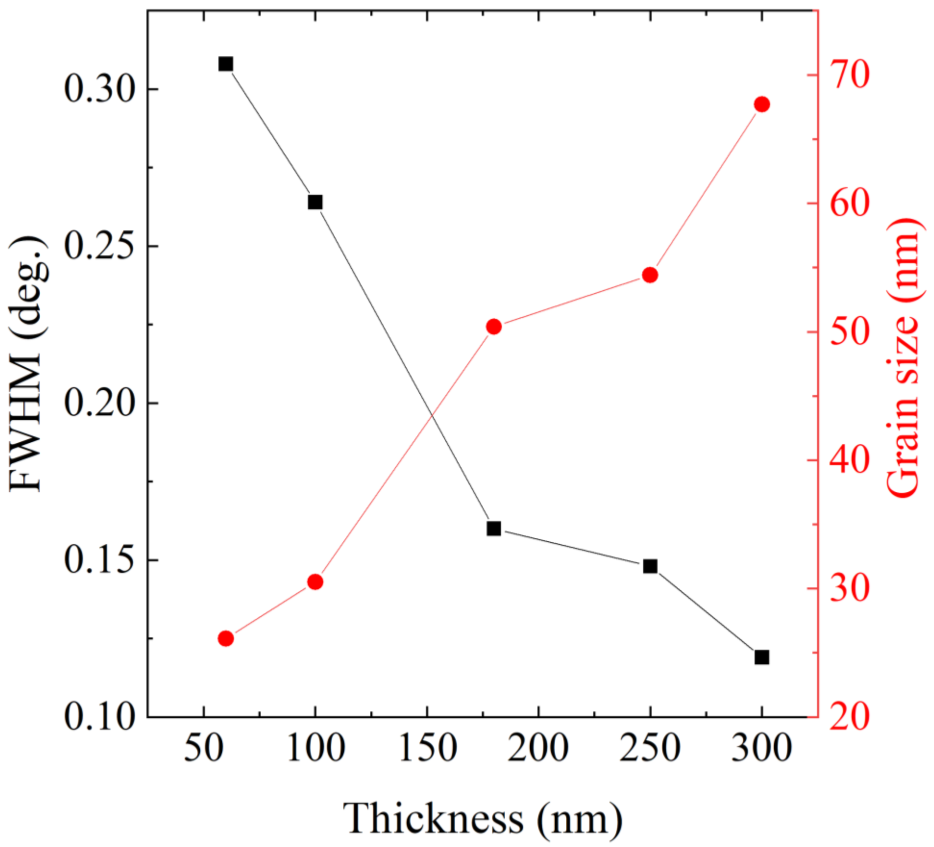

Supplementary Fig. 1. Thickness dependent grain size. FWHM of XRD peaks and grain size for CuI thin films with different thicknesses.

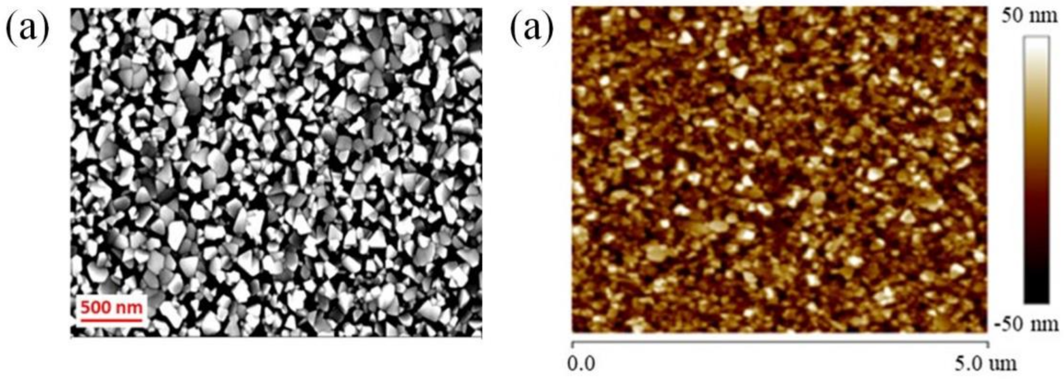

Supplementary Fig. 2. Surface morphology. SEM (**a**) and AFM (**b**) images for the as-grown CuI thin film.

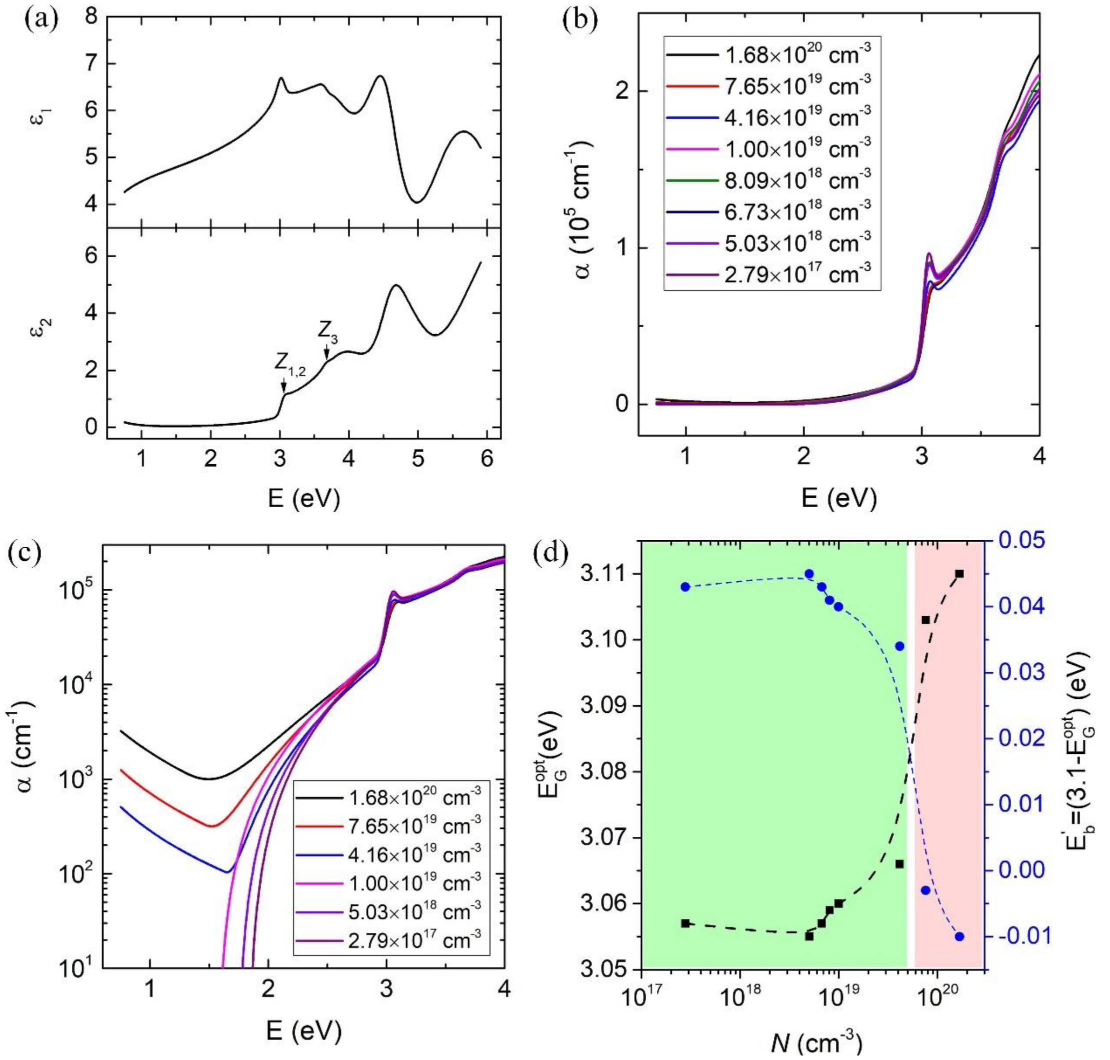

Supplementary Fig. 3. Dependence of optical properties on hole densities. a The real (ε_1, top panel) and the imaginary (ε_2, bottom panel) part of dielectric function of as-grown CuI thin film. **b** Absorption coefficient of CuI thin films with varying hole densities ranged from 10^{17}~10^{20} cm^{-3}, achieved by PTA in air at 100 ^{o}C for different durations. **c** Absorption coefficient of CuI thin films with different hole densities is scaled logarithmically. **d** Optical band gap $E_{\mathrm{G}}^{\mathrm{opt}}$ (left Y axis) derived from the zero crossing of $d\varepsilon_2/dE$ for CuI thin films with different hole densities, with the right Y axis denoting the quantity of $E_{\mathrm{b}}^{'} = 3.1 - E_{\mathrm{G}}^{\mathrm{opt}}$.

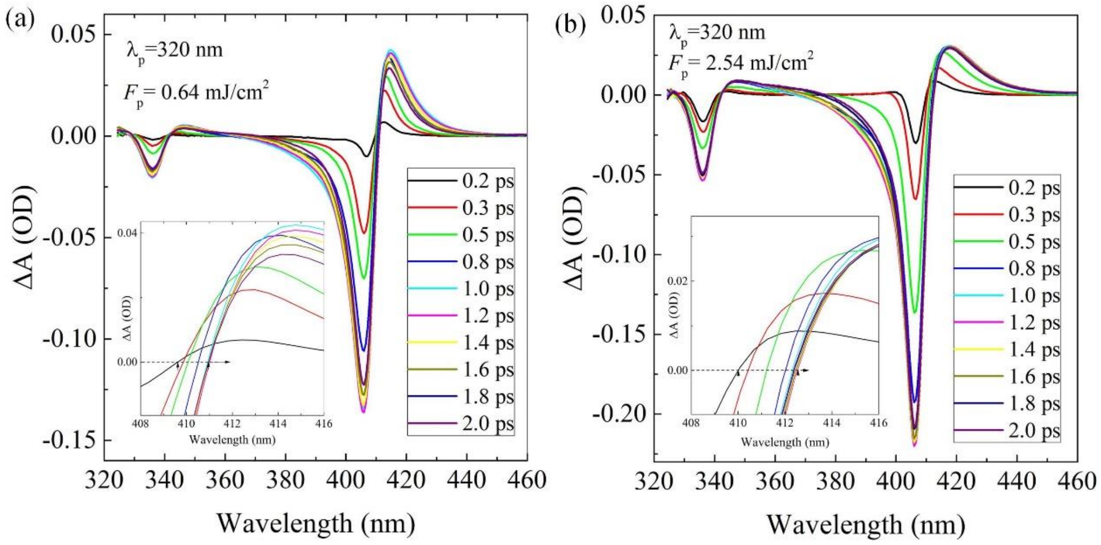

Supplementary Fig. 4 TA spectra as function of pump fluence and time delay. Transient absorption spectra of as-grown CuI thin film at different time delay range from 0.2 ps to 2 ps under different pump fluences of 0.64 mJ/cm^2 (**a**) and 2.54 mJ/cm^2 (**b**), respectively. Inset: TA spectra zoomed-in the PIA region, with vertical arrows indicating the transition energy between PIA and PB.

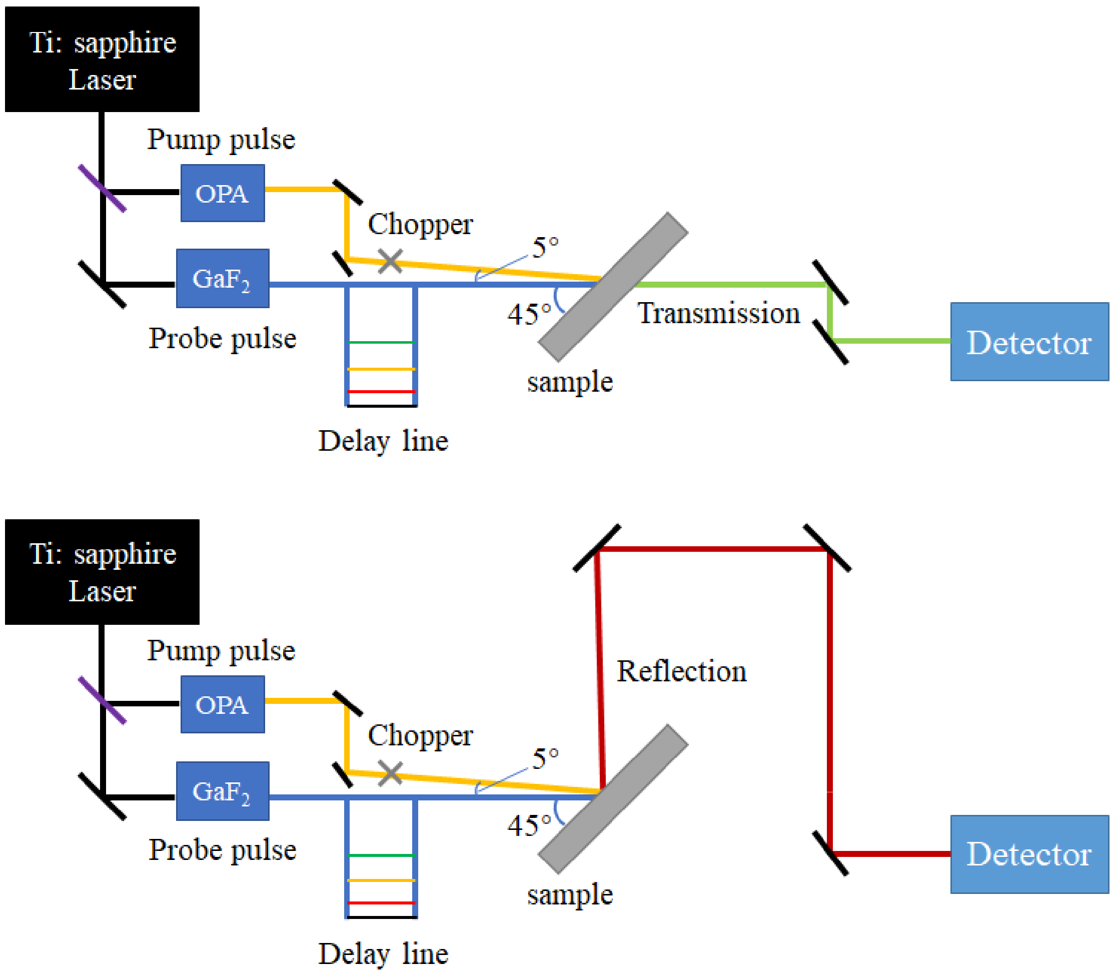

Supplementary Fig. 5. The schematic diagram of transient transmission (up panel) and transient reflection (low panel) measurements. The incident angle of probe beam is 45°.

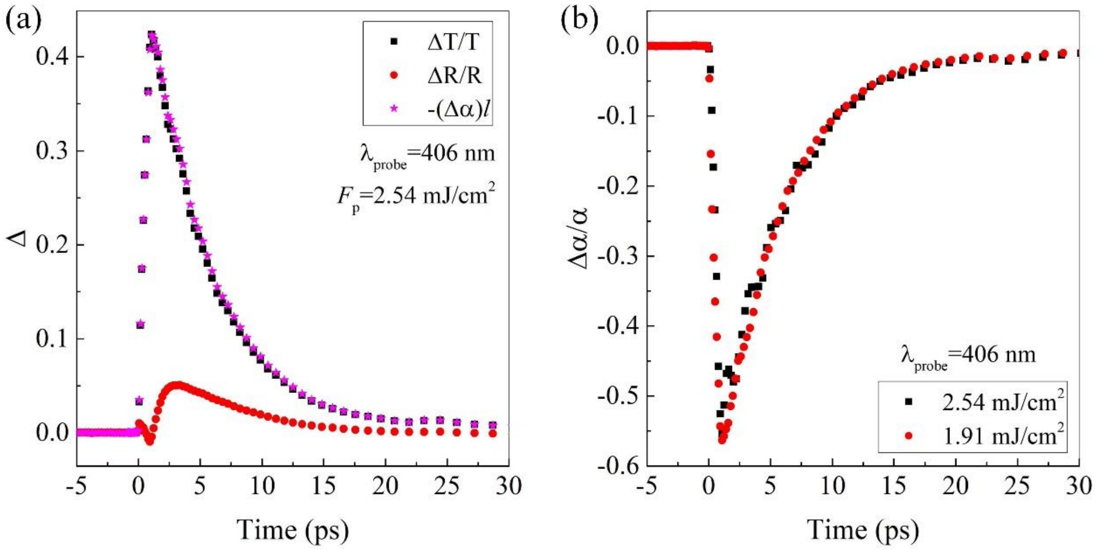

Supplementary Fig. 6 Relative change in transmission, reflection and absorption coefficient. **a** the transmission change $\Delta T/T$, reflection change $\Delta R/R$, and $(-\Delta\alpha)l$ of CuI thin film (N~1×10^{19} cm^{-3}) as function of time delay with λ_{probe}=406 nm, λ_p=320 nm, F_p=2.54 mJ/cm^2. **b** The corresponding absorption coefficient change $\Delta\alpha/\alpha$ at λ_{probe}=406 nm under pump fluences of 2.54 mJ/cm^2 and 1.91 mJ/cm^2.

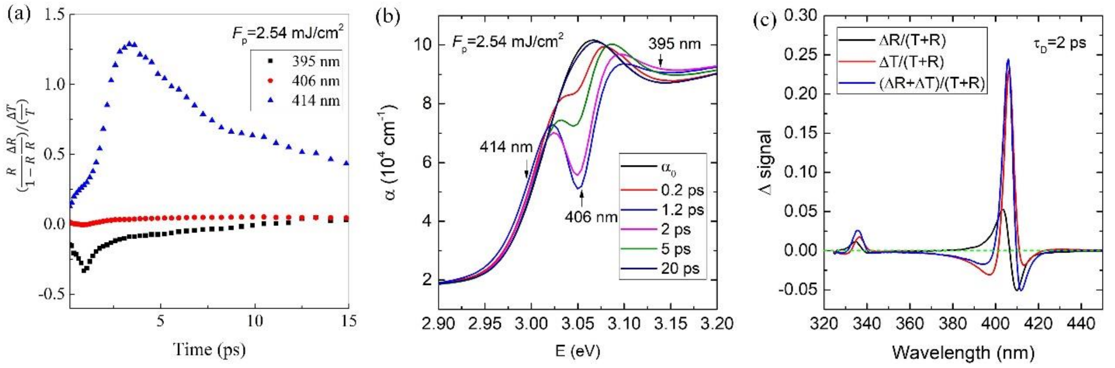

Supplementary Fig. 7 Transient optical properties. **a** $(\frac{R}{1-R}\frac{\Delta R}{R})/(\frac{\Delta T}{T})$ as function of time delay at different wavelengths (i.e., 395 nm, 406 nm, 414 nm) under pump fluence of 2.54 mJ/cm^2 ($\lambda_p = 320\ nm$). **b** The corresponding absorption coefficient α of as-grown CuI thin film at different time delays, with α_0 denoting the absorption coefficient without pump excitation. **c** Transient absorption and reflection spectra (λ_p=320 nm, F_p=2.54 mJ/cm^2) at a time delay of 2 ps weighted by the sum of transmission (T, simulated by SE) and reflection (R, simulated by SE) under incident angle of 45°.

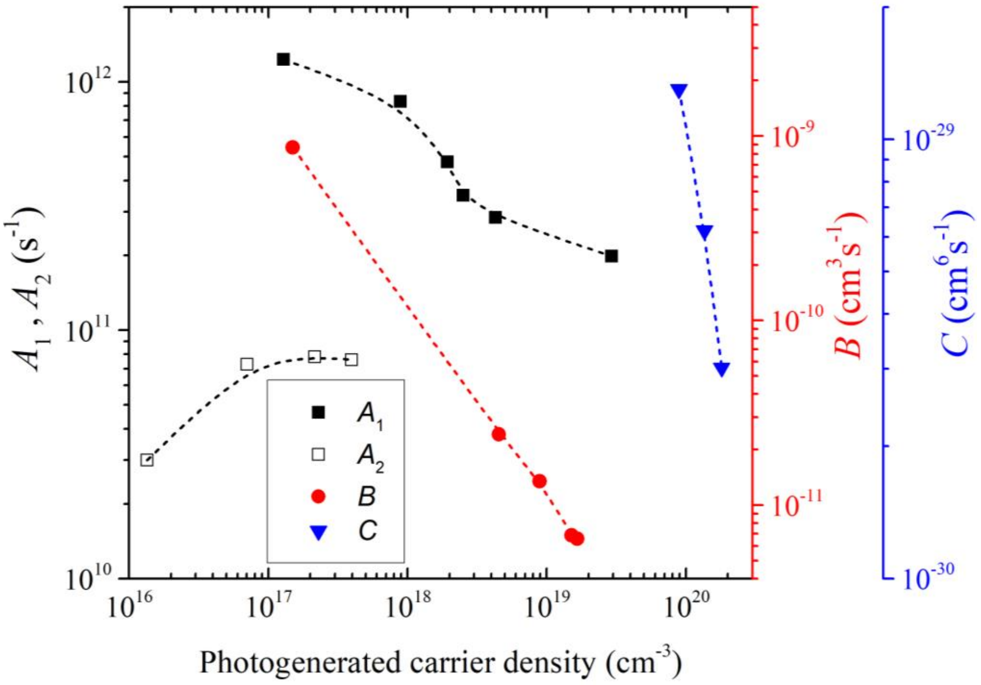

Supplementary Fig. 8. Photogenerated Carrier density-dependent recombination coefficients. SRH recombination coefficient A_1, exciton recombination coefficient A_2, bimolecular recombination coefficient B, and Auger recombination coefficient C are extracted from the as-grown CuI thin film.

Supplementary Table 1. Time constants for the bleach dynamics with different pump fluence F_p (λ_p=320 nm) probed at 395 nm.

F_p (mJ/cm^2)	0.13	0.64	1.27	1.91
τ (ps)	1.0	1.5	3.5	3.4

Supplementary Table 2. Time constants for the bleach dynamics with different pump fluences probed at 406 nm, with $\lambda_p = 520$ nm

F_p (mJ/cm^2)	0.26	0.78	1.91	2.60
τ (ps)	0.69	1.10	1.87	2.65

Supplementary Table 3 Recombination coefficients (*B* and *C*) for the CuI thin films with different treatments.

recombination coefficient	as-grown	PTA in air	PTA in I_2
B/ cm^3/s	1.30×10^{-11}	0.88×10^{-11}	2.06×10^{-11}
C/ cm^6/s	7.23×10^{-30}	16.4×10^{-30}	4.94×10^{-30}

Supplementary Note 1: Derivation of the transient absorption coefficient change Δα

In our transient reflection measurements, the incident angle of probe beam is 45°, as shown in below graph. For simplicity, we neglected the corresponding substrate and the reflection at the bottom surface of film.

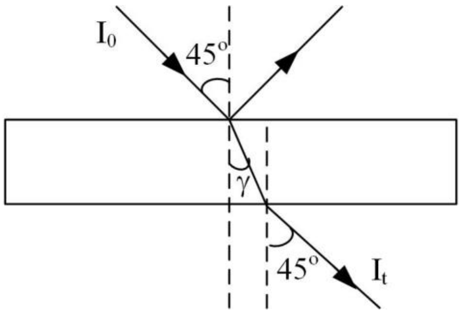

The thickness of thin film is l_0, and the intensity of incident beam and the transmitted beam are I_0 and I_t, respectively. The intensity of transmitted beam I_t can be written as,

$$I_t = I_0(1-R)e^{-\alpha l} ,$$

where R is the reflectance at the film surface, α is the absorption coefficient, l is the path length of the light beam in the film ($l = l_0/cos\gamma$, with γ the refraction angle). Taking the total differentiation of I_t, one can get

$$\Delta I_t = I_0(1-R)e^{-\alpha l}(-\Delta\alpha)l + I_0 e^{-\alpha l}(-\Delta R) = I_t(-\Delta\alpha)l + I_0 e^{-\alpha l}(-\Delta R).$$

Therefore,

$$\frac{\Delta I_t}{I_t} = (-\Delta\alpha)l - \frac{\Delta R}{1-R} ,$$

or,

$$(-\Delta\alpha)l = \frac{\Delta I_t}{I_t} + \frac{\Delta R}{1-R} = \frac{\Delta I_t}{I_t} + \frac{R}{1-R}\frac{\Delta R}{R} = \frac{\Delta T}{T} + \frac{R}{1-R}\frac{\Delta R}{R} ,$$

where ΔT and ΔR are the changes in transmission and reflection, respectively.
